# Evaluation of Clinical Findings and Treatment Results of Coronavirus Disease 2019 (COVID-19) in Pediatric Cancer Patients: A Single Center Experience

**DOI:** 10.3389/fped.2022.848379

**Published:** 2022-03-10

**Authors:** Orkun Tolunay, Ümit Çelik, Ilknur Arslan, Bugra Tutun, Merve Özkaya

**Affiliations:** ^1^Department of Pediatrics, University of Health Sciences Adana City Training and Research Hospital, Adana, Turkey; ^2^Department of Pediatric Infectious Diseases, University of Health Sciences Adana City Training and Research Hospital, Adana, Turkey

**Keywords:** child, COVID-19, cancer, results, SARS-CoV-2

## Abstract

**Objective:**

The objective of the study is to evaluate the clinical findings and treatment results of COVID-19 in pediatric cancer patients.

**Study design:**

The study was designed as a single-center retrospective observational study. Pediatric cancer patients with COVID-19 admitted to the University of Health Sciences Adana City Training and Research Hospital pediatric COVID wards from October 2020 to October 2021 were included. Clinical data and demographic characteristics were retrieved from patient files.

**Results:**

A total of 45 pediatric cancer patients diagnosed with COVID-19 were included in the study. The mean age of the patients was 8.68 ± 5.16 years (range 1.5–17.5), 62.2% were men, 37.8% were Turkish citizens, and 62.2% were Syrian refugees. A total of 41 patients (91.1%) had leukemia/lymphoma, while 4 (8.9%) had solid tumors. The most common symptoms were fever (66.7%), respiratory (35.6%), and gastrointestinal symptoms (17.8%). Disease severity was evaluated as mild in 46.7%, moderate in 44.4%, and severe in 8.9% of patients. Patients presented with lymphopenia (88.9%), thrombocytopenia (73.3%), anemia (71.1%), and neutropenia (62.2%). Mean hospital length of stay was 15.18 ± 10.34 (range 6–62) days overall and 9.5 ± 2.39 (range 2 to 28) days in the PICU. Intensive care unit admission rate was 8.9%, and mortality rate was 4.4%. Median viral shedding period was 21 days (range 7–52).

**Conclusions:**

Our study reveals that the mortality rate, length of hospital stay, and the need for intensive care of pediatric cancer patients with COVID-19 are higher than those of healthy children. Prospective studies with larger sample sizes are needed to further evaluate the clinical findings and treatment results of COVID-19 in pediatric cancer patients.

## Introduction

The coronavirus disease 2019 (COVID-19) has affected the whole world for the last 2 years. Specifically, it has a profound impact on patients with advanced age and underlying diseases (1). In children, pulmonary findings and disease severity increase with age (1, 2). Pediatric cancer patients are both affected by COVID-19 and face difficulty in receiving their treatment against cancer.

Studies of the last 2 years have shown that COVID-19 progresses with a more severe course in cancer patients, and morbidity and mortality rates are higher (3). The World Health Organization–China Joint Mission reported that the mortality rate of COVID-19 in China was 3.8% overall, while this rate was 7.6% in patients with cancer (3, 4). As with all COVID-19 studies, data are mostly based on adult studies. Data on pediatric cancer patients are limited. Some publications, especially from high-income countries presenting a small sample size, suggest that pediatric cancer patients might not have a higher mortality due to SARS-CoV-2 infection (5, 6).

This study, which was designed as a single-center retrospective observational study, was aimed to evaluate the clinical findings and treatment results of COVID-19 disease in pediatric cancer patients.

## Materials and Methods

The study was designed as a single-center retrospective observational study and conducted at the University of Health Sciences Adana City Training and Research Hospital (one of the main centers in the region treating pediatric COVID-19 patients). Pediatric cancer patients admitted to the pediatric COVID-19 wards with COVID-19 from October 2020 to October 2021 were included. Clinical data, demographic characteristics, and laboratory-imaging findings were retrieved from electronic medical files. COVID-19 diagnosis was obtained with real-time reverse transcription polymerase chain reaction (RT-PCR) assay (nasopharyngeal swab) for SARS-CoV-2. The COVID-19 treatment of the patients was arranged according to the Republic of Turkey Ministry of Health COVID-19 Guidelines (7). Discharge criterion was clinical symptom improvement. Providing a control PCR negative result was not required. Pediatric patients with ongoing cancer treatment were included in the study. Patients were classified according to the “hospital-based clinical staging system for COVID-19” (8, 9).

- Asymptomatic (Stage A): Patients with no signs or symptoms of COVID-19.
- Mild infection (Stage B): Patients with mild symptoms including fever, gastrointestinal symptoms, and upper respiratory tract infection symptoms. Patients with no evidence of pneumonia.
- Moderate infection (Stage C): Patients with hypoxia at rest (oxygen saturation <93%) or presence of pneumonia. Patients with no need for intensive care unit admission.
- Severe infection (Stage D): Patients requiring intensive care unit admission for pneumonia or any of the following: 1. Respiratory rate >30 breaths/min; 2. PaO_2_/FiO_2_ <300; 3. Lung involvement >50% on imaging within 24–48 h 4. Mechanical ventilation, septic shock, or multiorgan dysfunction.

### Ethical Approval

The study was approved by the University of Health Sciences Adana City Training and Research Hospital Clinical Research Ethics Committee (12.02.2021, Meeting number: 94, Decision no: 1657). An informed consent was waived because of the retrospective nature of the study.

### Statistical Analysis

Statistical analyses were conducted using the SPSS statistical software version 20 (IBM Corp., Armonk, NY, USA). Descriptive statistics of the numerical parametric data were calculated as mean ± standard deviation; non-parametric data were calculated as median and interquartile range (IQR), categorical variables were expressed as a percentage (%), an χ2 test was used for the comparison of categorical variables, independent samples *t*-test and Mann–Whitney *U*-test were used to compare the numerical variables between groups. The one-way ANOVA is used to compare the means of more than two groups when there is one independent variable and one dependent variable. Significant differences are indicated as a *p*-value <0.05.

## Results

A total of 45 pediatric cancer patients with COVID-19 disease who were treated in our clinic were included in the study. The mean age of patients was 8.68 ± 5.11 years (median: 7.5, IQR: 4.3–13.5); 37.8% were females, 62.2% were males, 37.8% were Turkish citizens, and 62.2% were Syrian refugees (Table 1). Age distribution was as follows: 26.7% were 5 years and younger, 42.2% were between 6 and 12 years, and 31.1% were 13 years and older. A total of 41 patients (91.1%) had leukemia/lymphoma, while 4 (8.9%) had solid tumors (Table 2).

**Table 1:** Demographic and clinic characteristics of the patients.

**Characteristics**	**Leukemia/** **lymphoma (*n* = 41, 91.1%)**	**Solid tumors (*n* = 4, 8.9%)**	**Overall (*n* = 45, 100%)**	***p*-Value**
Gender (*n*, %)				0.626
Female Male	15, 36.6% 26, 63.4%	2, 50% 2, 50%	17, 37.8% 28, 62.2%	
Age (years) ^*^	7.5 (4.3–12.5)	10 (3.9–16.5)	7.5 (4.3–17.5)	0.570
Age group				0.665
0–5 years 6–12 years >13 years	11, 26.8% 18, 43.9% 12, 29.3%	1, 25% 1, 25% 2, 50%	12, 26.7% 19, 42.2% 14, 31.1%	
Disease severity				0.806
Mild Moderate Severe	19, 46.3% 18, 43.9% 4, 9.8	2, 50% 2, 50% –	21, 46.7% 21, 44.4% 4, 8.9%	
Hospital length of stay (days)^*^	13 (8.5–17)	13 (10.5–18.5)	12.5 (9.2–17)	0.154
PICU length of stay (days)^*^	4 (2, 25–22, 25)	–	4 (2, 25–22, 25)	–
PICU requiring (n, %)	4, 9.8%	–	4, 8.9%	–
Steroid (n, %)	8, 19.5%	–	8, 17.8%	–
Favipiravir	8, 19.5%	–	8, 17.8%	–
Hydroxychloroquine	1, 2.4%	–	1, 2.2%	–
Chest CT (*n*, %) Normal Abnormal ^**^	12, 57.1% 9, 42.9%	3, 100% 0	15, 62.5% 9, 37.5%	0.266
Comorbidities (*n*, %) ^***^	4, 9.8%	–	4, 8.9%	–

* *Median and interquartile range*.

** *Ground-glass opacities, nodular lesions, pleural effusion, consolidation*.

*** *Diabetes mellitus (n = 1), cerebral venous sinus thrombosis (n = 1), dilated cardiomyopathy (n = 1), right atrial thrombus (n = 1)*.

**Table 2:** Cancer type of the patients.

**Cancer type**	**(*n*, %)**
**Leukemia/lymphoma**	**41, 91.1%**
Acute lymphocytic leukemia	29, 64.4%
Acute myeloid leukemia	8, 17.8%
Non-Hodgkin's lymphoma	3, 6.6%
Hodgkin's lymphoma	1, 2.2%
**Solid tumors**	**4, 8.9%**
Ewing sarcoma	1, 2.2%
Rhabdomyosarcoma	1, 2.2%
Neuroblastoma	1, 2.2%
Osteosarcoma	1, 2.2%

The most common symptoms were fever (66.7%), respiratory symptoms (35.6%), and gastrointestinal symptoms (17.8%) (Table 3). All patients received antibiotic therapy, 20% were administered COVID-19 therapy (favipiravir, hydroxychloroquine), and 17.8% received steroid therapy for COVID-19. The need for blood product (red blood cells, fresh frozen plasma, or platelets) transfusion was 64.4%. Six (13.3%) patients received low-molecular-weight heparin, and 29 (66.4%) patients received granulocyte colony-stimulating factor (G-CSF) during their hospitalization. No blood culture-proven bloodstream infection was detected in the follow-up of the patients.

**Table 3:** Clinical manifestations of the patients.

**Characteristics**	(%)^*^
Fever (%)	66.7%
Respiratory symptoms (%) Cough (%) Shortness of breath	35.6% 35.6% 8.9%
Gastrointestinal symptoms (%)	17.8%
Vomiting Nausea Diarrhea Abdominal pain	4.4% 4.4% 6.6% 2.2%
Loss of appetite	4.4%
Weakness (%)	6.6%
Flu-like symptoms (%)	4.4%
Sore throat (%)	2.2%

* *A patient may have more than one symptom*.

Hospital-based clinical staging system for COVID-19 classification was as follows: 21 patients (46.7%) had mild disease, 20 patients (44.4%) had moderate, and 4 patients (8.9%) had severe disease (7, 8). The mean time between positive SARS-CoV-2 PCR and the first negative result (viral shedding) was 20.32 ± 9.52 (median: 21, IQR: 13–27) days (31 patients have viral shedding data). The patient with the highest viral shedding time (52 days) was diagnosed with MIS-C during his hospitalization; IVIG and steroid treatments were also administered. The mean hospital length of stay was 15.18 ± 10.34 (median: 13, IQR: 9–17) days overall and 9.5 ± 12.39 (median: 4, IQR: 2.25–22.25) days in the PICU. Intensive care unit admission rate was 8.9%, oxygen support was required in 35.6% of the children, 3 patients (6.7%) required high-flow nasal cannula oxygen therapy, and 2 patients (4.4%) required mechanical ventilation.

Despite treatments, two (4.4%) patients died due to multiorgan failure. One of them was a patient with acute lymphocytic leukemia (ALL) who had undergone bone marrow transplant 1.5 years before and relapsed 1 month ago, while the other patient was diagnosed with acute myeloid leukemia (AML) 3 months ago. Both patients had received chemotherapy about 1 month ago. Both patients were followed up with invasive mechanical ventilation in the pediatric intensive care unit. In addition to G-CSF, broad-spectrum antibiotics, and antifungal treatments, the patients were also given favipiravir treatment for COVID-19. Four patients had comorbidities (not related to COVID-19) besides cancer, but no mortality was observed in these patients (Table 1).

**Figure 1:**
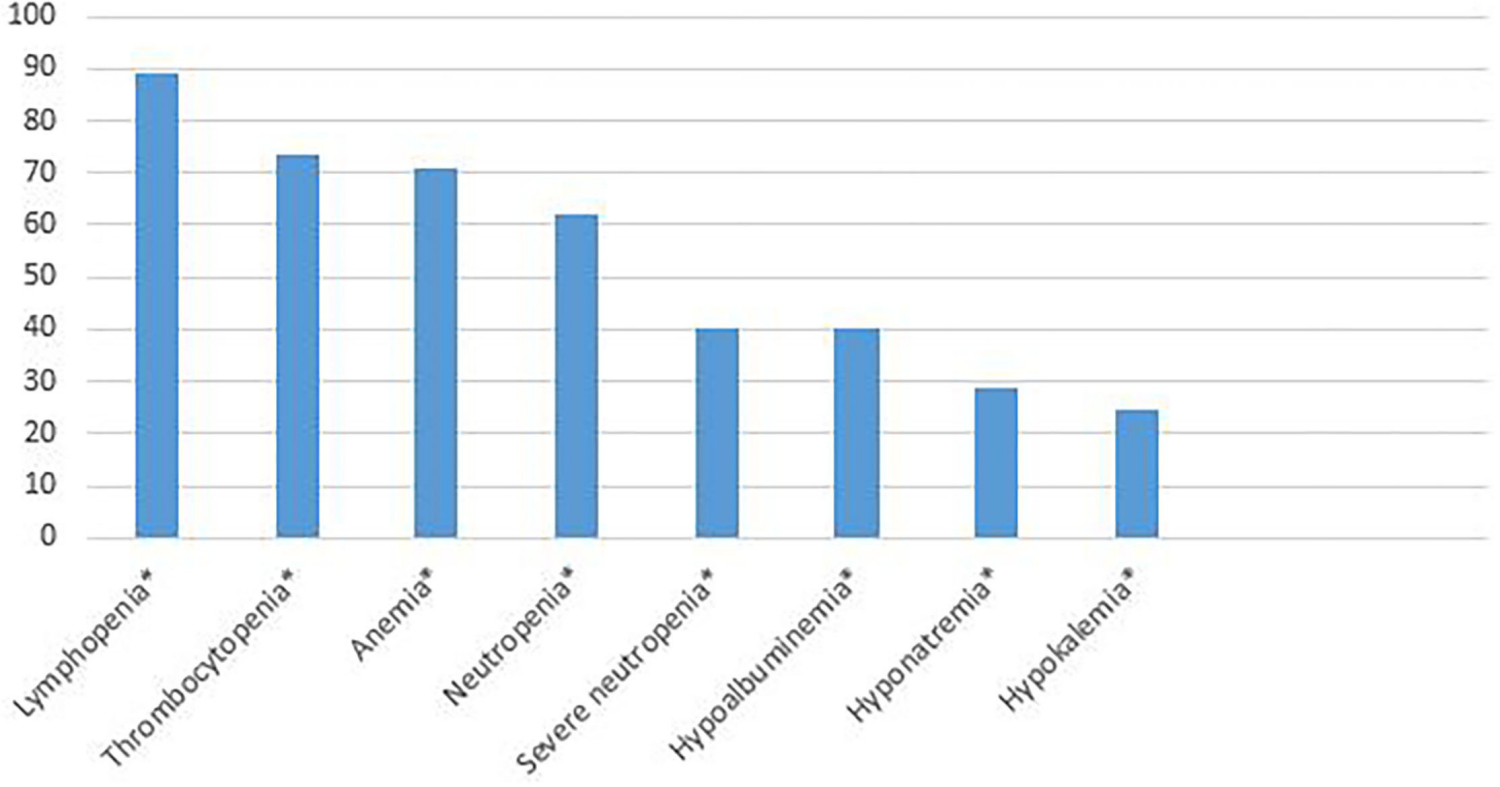
The percentages of laboratory anomalies at admission. ^*^Lymphopenia (absolute lymphocyte count < 1.5 × 10^9^/L), thrombocytopenia (platelet count < 150.000 × 10^9^/L), anemia (less than the fifth percentile for age), neutropenia (absolute neutrophil count < 1.5 × 10^9^/L), severe neutropenia (absolute neutrophil count <0.5 × 10^9^/L), hypoalbuminemia (<35 g/L), hyponatremia (< 135 mmol/L), hypokalemia (<3.5 mmol/L).

Thoracic computed tomography imaging was performed in 24 patients (53.3%). Significant radiologic findings were detected in 9 of them (9/24, 37.5%). Ground glass opacities, nodular lesions, pleural effusion, and consolidation were among the pathologies detected. At admission, anemia (less than the fifth percentile for age) (10), lymphopenia (wabsolute lymphocyte count <1.5 × 10^9^/L), neutropenia (absolute neutrophil count <1.5 × 10^9^/L), severe neutropenia (absolute neutrophil count <0.5 × 10^9^/L), thrombocytopenia (platelet count <150.000 × 10^9^/L), hyponatremia (<135 mmol/L), hypokalemia (<3.5 mmol/L), and hypoalbuminemia (<35 g/L) were detected in 71.1, 88.9, 62.2, 40, 73.3, 28.9, 24.4, and 40% of the patients, respectively (Figure 1). Of the patients, 91.1% had CRP levels >5 mg/L, 77.8% had ESR levels >20 mg/h, 91.1% had procalcitonin levels >0.1 μg/L, 88.4% had ferritin levels >336 μg/L, and 84.6% had D-dimer levels >550 μg/L. Laboratory results are shown in Table 4.

**Table 4:** Laboratory test results of the patients.

**Characteristics**	**On admission**	**On discharge**
White blood cells, 10^9^/L Median (IQR)	1.8 (0.7–3.6)	5.9 (4.1–11.05)
Neutrophil count, 10^9^/L Median (IQR)	0.8 (0.1–2.55)	3.55 (1.82–6.7)
Lymphocyte count, 10^9^/L Median (IQR)	0.5 (0.2–0.8)	1.4 (0.7–2.6)
Platelets, 10^9^/L Median (IQR)	72 (24–159)	197 (76.5–321.5)
Hemoglobin, g/Dl Median (IQR)	9.5 (7.8–11.4)	10.7 (9.6–11.8)
CRP, mg/L (0–5) Median (IQR)	58.8 (9.7–102.7)	6.7 (11.5–17.55)
Procalcitonin, μg/L (0–0.065) Median (IQR)	0.37 (0.13–1.01)	0.1 (0.07–0.27)
Erythrocyte sedimentation rate, mm/h (0–20) Median (IQR)	51 (32.5–78.5)	28 (16.5–54.5)
Ferritin, μg/L (23.9–336) Median (IQR)	1,171 (579.5–1,796)	1,200 (600.25–2,192)
LDH, U/L (110–295) Median (IQR)	246 (192–373.5)	300 (226.5–374.5)
D-dimers, μg/L (150–550) Median (IQR)	1,140 (580–3,105)	710 (350–2.790)
Albumin, g/L (35–55) Median (IQR)	36.1 (32.1–38.25)	36.45 (32.8-39.57)
Na, mmol/L (136–146) Median (IQR)	138 (135–139)	138 (136-139)
K, mmol/L (3.5–5.5) Median (IQR)	3.98 (3.51–4.33)	4.23 (4-4.68)

A statistically significant difference was found between mortality and disease severity (*p* < 0.001). No statistically significant differencew was found between mortality and sex, age, nationality, disease severity, hospital length of stay, cancer type (leukemia/lymphoma or solid tumors), neutropenia, severe neutropenia, lymphopenia, thrombocytopenia, anemia, hypoalbuminemia, hyponatremia, hypokalemia, and high levels of CRP, ESR, and procalcitonin.

No statistically significant difference was found between cancer type (leukemia/lymphoma or solid tumors) and sex, age, nationality, disease severity, mortality, need for intensive care, hospital length of stay, neutropenia, severe neutropenia, lymphopenia, thrombocytopenia, anemia, hypoalbuminemia, hyponatremia, hypokalemia, and high levels of CRP, ESR, and procalcitonin.

## Discussion

It has been clearly demonstrated in the past 2 years that the Covid-19 disease progresses milder in children compared with adults (1, 11). However, children may have complications, such as the MIS-C syndrome, which occurs after 4–6 weeks of the COVID-19 disease (2, 11). It has also been observed during the course of the pandemic that regardless of whether they are children or adults, patients with comorbidities have the disease more severely (12). Primarily through the direct infection and by delaying cancer treatments, COVID-19 increases morbidity and mortality in cancer patients (3, 4, 13). It has been clearly demonstrated that mortality and morbidity of COVID-19 is high in adult cancer patients, but such clear information is not available for pediatric cancer patients. Some studies advise that COVID-19 progresses like other viral infections in pediatric cancer patients, and therefore, cancer treatment should not be delayed (14, 15). Common features of such studies are that they are conducted with a small number of patients and include asymptomatic or outpatient pediatric cancer COVID-19 patients (14, 15). Our study includes symptomatic COVID-19 pediatric cancer patients whose chemotherapy or radiotherapy were interrupted due to COVID-19.

In studies on COVID-19 comprising previously healthy children, it has been shown that COVID-19 can be seen at any age, including the neonatal period (11, 16). Although our study included only pediatric cancer patients, it covers a wide age distribution between 1.5 and 18 years. Although different results can be observed when COVID-19 patients are evaluated in terms of gender, male predominance is mostly determined in pediatric and adult studies (11, 16–18). Similar results are obtained in pediatric cancer patients (3, 14, 19). Similar to the literature, the male/female ratio was 1.6 in our study.

While the first symptoms of pediatric patients at the time of admission were cough and fever, similar results were obtained in studies with cancer patients (3, 14, 16, 19). In the study of Dong et al. cough was the most common symptom with 48.5%, followed by fever with 41.5% (17). Similar results were obtained in the study of Yilmaz et al. (16). The most common symptoms in our study were fever and cough.

When the severity of COVID-19 in pediatric patients was evaluated, it was found that children mostly experienced COVID-19 as asymptomatic and mild (1, 16). In the study of Yilmaz et al. the rate of asymptomatic patients of previously healthy children was determined as 56%, and only 2.9% of the patients were considered as severely ill (16). Studies conducted in different centers and countries reported the rate of severe COVID-19 cases to be between 0.6 and 2.5% (11, 17, 20, 21). In our study, the rate of severe disease was detected as 8.9%, which is above the results available in the literature.

The need for intensive care in pediatric patients is also less than in adult patients (11). In the study conducted by Bialek et al. on 123 patients, none of the patients required intensive care (21). In the study of Yilmaz et al. (16) the need for intensive care was determined as 2.9%, while this rate was found as 1.8% in the study of Yang et al. (20). In the study of Yayla et al. although comorbidity was detected in 10% of the patient group, the need for intensive care was 1.4% (22). In the studies of Antúnez-Montes et al. while the rate of children with immunodeficiency was 4.4%, the need for intensive care increased to 11.5% in these patients (23). Similarly, while the rate of patients with a history of chemotherapy was 3.4%, the need for intensive care was 7.7% (23). Due to the special conditions of our patient group, the rate of intensive care requirement was 8.9%.

In studies conducted on hospitalized pediatric patients with COVID-19, mortality rates were found to be low (18, 24). While no mortality was detected in the study of Alattas and Bialek et al. (18) it was found to be 0.19% in the study of Bialek et al. (21), Moreira et al. (24). In our study, the mortality rate was found to be 4.4%, being considerably higher than the existing studies. The results of mortality rates in pediatric cancer patients might be confusing specifically in studies that are conducted on a small sample size. While no mortality was found in the studies of de Rojas et al. (5) the mortality rate was found to be 28% in the studies of Jabor et al. (6).

In their study on adult patients, Thai et al. found that the median length of stay in the hospital due to COVID-19 was 10–13 days (25). In studies conducted with pediatric patients, Yayla et al. found that the median length of stay in the hospital was 10 days (min–max: 10–41), while Rabha et al. found it to be 4 days (min–max: 2–11) and Yilmaz et al. determined it to be 4 days (min–max: 1–19) (16, 25, 26). In our study, the median length of hospital stay was 13 days (min–max: 6–52).

In adult studies, solid tumors (breast cancer, lung cancer) and hematological malignancies have been pointed out as groups at high risk of COVID-19 death and intensive care admission (27, 28). In subanalyses of cancer type, lung cancer and hematological malignancies were identified as groups at high risk of COVID-19 death with no recent chemotherapy record. It is stated that hematological malignancies often cause immunological deficiency, and lung cancer leads to these results by impairing lung function (27, 28). In studies on pediatric cancer patients, no significant relationship was found between cancer type (leukemia/lymphoma or solid tumors) and mortality, disease severity, and the need for intensive care (29). Similarly, no significant relationship was found between cancer type and treatment outcomes in our study.

Although different results on viral shedding through respiratory tract are prevalent, the 14-day isolation recommendation for COVID-19 patients still remains valid (30). While viral shedding was found to be 11.1 ± 5.8 days in the study of Hu et al. this period was found to be 17.6 ± 6.7 days in the study of Han et al. (31, 32). While it is not yet proven for SARS-CoV-2, prolonged viral shedding (>21 days) has been shown in pediatric cancer patients (2, 33, 34). In our study, the duration of viral shedding was found to be 21.1 ± 11.02 days, which is longer compared with previously healthy children.

According to the International Organization for Migration (IOM), refugees are more vulnerable than others in contracting contagious diseases, especially SARS-CoV-2 because of personal, social, infrastructural, and health factors (35). Statistical data on the impact of COVID-19 on refugees are scarce, but a growing number of literature reveals that refugees, an already vulnerable group, have further reduced their wellbeing during the pandemic (36). Turkey is among the top five countries hosting refugees (35). As in the rest of the world, the socioeconomic situation of refugees in our country is likely to be worse than that of the local people. However, there is no distinction between the citizens of the Republic of Turkey and refugees in the provision of health services in our country. Due to the retrospective nature of our study, we cannot clearly state the difference in socioeconomic level between the two groups. However, in our study, no difference was found in the COVID-19 treatment outcomes between the citizens of the Republic of Turkey and the refugees.

The main limitation of the study is that this was a retrospective, single-center study with a relatively small sample size. Another limitation is that our hospital is a reference center, and pediatric cancer patients with COVID-19 have been referred from different centers, so the chemotherapy regimens of the patients are not exactly known. Since a study is being conducted on previously healthy patients with COVID-19 in our clinic, and the use of the data of these patients in our study may cause ethical problems, we could not create a control group, even if we wanted to. Therefore, we had to compare the results of our patient group with the results of similar studies in the literature. Prospective studies with larger sample sizes are needed to further evaluate the clinical findings and treatment results of COVID-19 in pediatric cancer patients.

### Conclusions

In conclusion, our study reveals that the mortality rate, length of hospital stay, and the need for intensive care of pediatric cancer patients with COVID-19 are higher than those of previously healthy children. Compared with healthy children, children with comorbidities, such as cancer, are more vulnerable to COVID-19. Until now, studies conducted on the effects of COVID-19 on pediatric cancer patients had a small sample size with presenting conflicting results. Therefore, prospective and welldesigned studies with large patient numbers are needed.

## Data Availability Statement

The original contributions presented in the study are included in the article/supplementary material, further inquiries can be directed to the corresponding author/s.

## Ethics Statement

The study was granted ethical approval by the University of Health Sciences, Adana City Training and Research Hospital Clinical Trials Ethics Committee.

## Author Contributions

OT, ÜÇ, IA, BT, and MÖ: substantial contributions to the conception or design of the work, acquisition, analysis, interpretation of data for the work, drafting the work, revising it critically for important intellectual content, final approval of the version to be published, and agreement to be accountable for all aspects of the work in ensuring that questions related to the accuracy or integrity of any part of the work are appropriately investigated and resolved. All authors contributed to the article and approved the submitted version.

## Conflict of Interest

The authors declare that the research was conducted in the absence of any commercial or financial relationships that could be construed as a potential conflict of interest.

## Publisher's Note

All claims expressed in this article are solely those of the authors and do not necessarily represent those of their affiliated organizations, or those of the publisher, the editors and the reviewers. Any product that may be evaluated in this article, or claim that may be made by its manufacturer, is not guaranteed or endorsed by the publisher.
